# Analysis of the Influence of Clear‐Sky Fluxes on the Cloud‐Type Mean Cloud Radiative Effects in the Tropical Convectively Active Regions With CERES Satellite Data

**DOI:** 10.1029/2024JD041525

**Published:** 2024-11-20

**Authors:** Kuan‐Man Xu, Moguo Sun, Yaping Zhou

**Affiliations:** ^1^ NASA Langley Research Center Hampton VA USA; ^2^ Analytical Mechanics Associates Inc./NASA Langley Research Center Hampton VA USA; ^3^ UMBC/NASA Goddard Space Flight Center Greenbelt MD USA

**Keywords:** cloud radiative effects, satellite data, flux by cloud type, tropical convective regions

## Abstract

Cloud radiative effects (CREs) and cloud‐type mean CREs depend upon how clear‐sky fluxes are computed over a large area: those of the immediate environment of clouds or the regional mean clear‐sky fluxes. Five convectively active regions in the Tropics, two over land (Africa and Amazon) and three over ocean (eastern and western Pacific and Atlantic), are selected to understand the influence of immediate environment of clouds on CREs. Fluxes derived from 19 years of high‐resolution CERES satellite data, categorized by cloud type, are utilized. The cloud types are classified based on the joint cloud top pressure and cloud optical depth distribution. For the entire tropical region, differences in cloud‐type mean CRE with regional mean and immediate environment clear skies range from −7.8 to 10.7 Wm^−2^ for shortwave (SW), 2.9 to 15.8 Wm^−2^ for longwave (LW), and 6.1 to 17.9 Wm^−2^ for net, respectively. The oceanic and Amazonia regions have negative (positive) SW (LW) CRE differences, typically 2–6 Wm^−2^ in SW but 7–10 Wm^−2^ in LW, whereas Africa has positive SW and LW CRE differences (typically 20–30 Wm^−2^, up to 40–50 Wm^−2^). The influence of immediate environment reduces the regionally averaged, that is, cloud‐type mean CREs weighted by cloud fractions, SW cloud cooling, and LW cloud warming in four of the five regions except for Africa. For Africa, it increases the SW cloud cooling and greatly reduces the LW cloud warming, resulting in net cloud cooling as in other regions instead of warming. The implications of these findings for observational and modeling studies are discussed.

## Key Points

- Examine the influence of immediate environment on the cloud‐type mean cloud radiative effects (CREs) in the Tropics with a CERES data set
- Use of regional mean clear‐sky flux causes too much longwave warming and insufficient shortwave cooling and net cloud warming over Africa
- CREs resemble each other more among five convectively active regions when considering the influence of immediate environment of clouds

## 1 Introduction

It is well known that clouds play an important role in the Earth's radiative energy budget and climate sensitivity (e.g., Hartmann et al., 1992; Ramanathan et al., 1989; Randall et al., 1984; Stephens, 2005). In general, clouds warm the atmosphere and surface through reduced outgoing infrared emission while they also cool the atmosphere and surface by reflecting solar radiation back to space. The net effects can be either cooling or warming, depending upon the cloud types. For example, low‐level clouds reflect much of the incoming solar radiation so that the underlying ocean absorbs very little solar energy (e.g., Randall et al., 1984). They have a net cooling effect because infrared emission from clouds is very close to that from the ocean surface. This means that longwave (LW) cloud radiative effect (CRE), which is defined as the difference in the top of the atmosphere (TOA) radiative flux between the clear and full skies (Ramanathan et al., 1989), is close to zero for low clouds. On the other hand, sum of shortwave (SW) CRE and LW CRE (hereafter, net CRE) of tropical anvil clouds can either be cooling or warming, depending upon how thick cloud optical depths of anvil clouds are. Because of the prevalence of deep convection and associated anvil clouds in the Tropics, it is generally regarded that the net CRE of the Tropics tends to approach zero (e.g., Hartmann & Berry, 2017; Hartmann et al., 1992; Kiehl, 1994).

The importance of understanding the CREs of different cloud types extends far beyond the Earth's radiative energy budget because the response of clouds to climate change and the enhancement/diminishment of climate change due to radiative imbalance caused by clouds, that is, cloud feedback, remain a major source of uncertainty for climate sensitivity in global climate models (e.g., Randall et al., 2007; Stephens, 2005; Vial et al., 2013). A recent study by Ceppi and Nowack (2021) concluded that global cloud feedback is positive and will amplify global warming from a cloud controlling factor analysis using observational and reanalysis data as well as model simulations. Another recent study by Raghuraman et al. (2023) showed a small positive cloud feedback and due to uncertainty a zero or negative feedback could not be ruled out. These analyses relied on the 20‐year Clouds and the Earth's Radiant Energy System (CERES; Wielicki et al., 1996) energy balanced and filled (EBAF) data product (Loeb et al., 2018, 2020), which provides the best estimates of CREs and radiative fluxes from satellite remote sensing. In particular, the CRE estimates consider the differences between satellite‐determined clear‐sky flux and cloud‐free grid‐mean flux (Loeb et al., 2020). The adjustment was designed to correct the overestimates of CREs resulting from more humid cloud‐free skies than clear skies outside of clouds, which may account for 10% of CREs (Sohn et al., 2010). However, it is noted that the adjustment to LW CRE over Africa and other land regions is minimal (Loeb et al., 2020). Owing to high land surface temperature over cloud‐free areas, the magnitude of LW CRE can be larger than that of SW CRE, resulting in net CRE being close to zero or even positive (see Boudala et al., 2022).

Another CERES data product called FluxByCldTyp (FBCT) can also produce the CRE estimates. This data product provides both daily and monthly daytime‐averaged cloud properties and radiative fluxes for each of 42 cloud types (Eitzen et al., 2017; Sun et al., 2022). Cloud types are defined by the joint distribution of effective cloud (“radiating” top) pressure ($p_{c}$) and cloud optical depth (τ) bins, following the International Satellite Cloud Climatology Project (ISCCP) classification (Rossow & Schiffer, 1999). So, CREs can be calculated from the cloud‐type mean radiative fluxes, which will be interchangeably called “CREs by cloud type” or “cloud type‐mean CREs.” CREs by cloud type are closely related to radiative‐transfer model‐based “cloud radiative kernels,” which describe the change in CRE per unit change of cloud fraction (Zelinka et al., 2012; Zhou et al., 2013) except for using observationally estimated radiative fluxes (Yue et al., 2016). The FBCT also resembles an ISCCP data product generated by Chen et al. (2000) using radiative transfer model‐calculated radiative fluxes with input from satellite cloud retrievals. The FBCT algorithm used in generating the FBCT data product is a refinement of the approach proposed by Cole et al. (2011) by improving the accuracy of radiative flux estimates. Further details of FBCT data product are briefly described in Section 2 and by Sun et al. (2022).

How the clear skies are chosen for a given cloud type in calculating the regionally averaged CREs and CREs by cloud type is the subject of this research. The original meaning of CRE is unambiguous, which is different from that of Ramanathan et al. (1989), because it refers to the radiative effect of a cloud without considering its environment. That is, CRE is the difference in the TOA radiative fluxes in the absence and presence of a cloud in a column with identical conditions (e.g., temperature, moisture, and aerosol profiles). When considering an area with both clear and cloudy skies, the CRE is defined as the difference in the TOA radiative flux between clear and full skies (Ramanathan et al., 1989). Clear skies are a part of the full sky and adjacent to a cloud or multiple clouds. Thus, the physics of horizontal differential heating is involved, which partially drives the cloud‐scale dynamics. For example, subsidence is enhanced as cloud updrafts strengthen, which enhances the differential heating between clear and cloudy skies and thus plays a major role in maintaining the convective self‐aggregation (Muller & Bony, 2015; Muller & Held, 2012; Pope et al., 2021).

**Figure 1:**
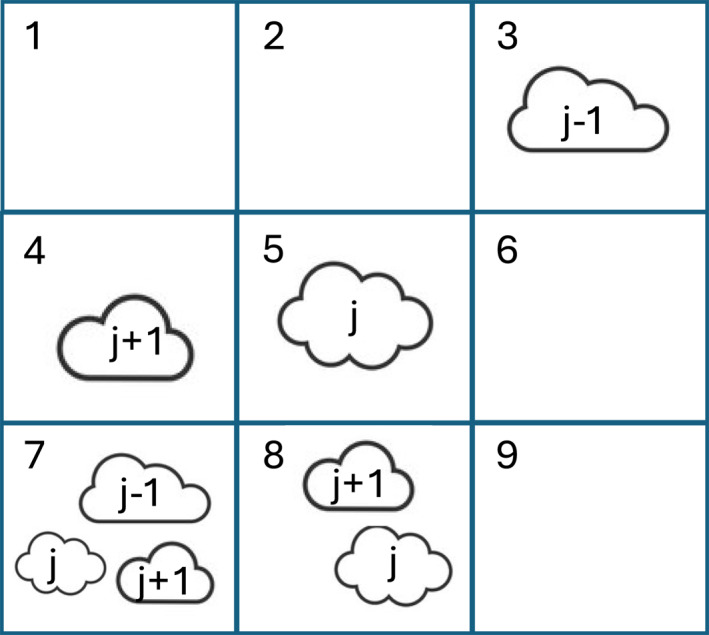
A schematic of a region composed of 9 grid boxes for illustrating how regional mean clear‐sky radiative fluxes are calculated by two different expressions. With Expression 1, clear‐sky flux for the region is computed from the average of the fluxes over clear‐sky portions of all 9 grids. With Expression 2, clear‐sky flux depends on cloud type and is cloud fraction‐weighted average of clear‐sky fluxes. For example, cloud type j‐1 is computed from Grids 3 to 7; cloud type j from Grids 5, 7 and 8; and cloud type *j*+1 from Grids 4, 7 and 8. There are multiple cloud types in Grids 7 and 8. Grids 1, 2, 6 and 9 do not contribute to the regional mean clear‐sky flux according to Expression 2, but they do according to Expression 1.

An appropriate choice of clear skies, the cloud‐free portion of 1° × 1° grid in FBCT data product, is critical in calculating regionally averaged CREs, particularly cloud‐type mean CREs. A simple choice for clear‐sky radiative flux is an average of clear‐sky radiative fluxes from all grids in a region for all cloud types, which may distort the real impact of clouds on the environments. In the real world, clear skies surrounding specific cloud types are not only spatially connected with clouds but also distinct from each other even within the same region. The rational for deriving a new clear‐sky flux expression is that clear skies should belong to the same grid in which a particular cloud type is produced. If a cloud type is absent, the clear‐sky flux over that grid should not contribute to CRE averaged over a domain composing of many 1° × 1° grids, which is called “a region” in this study (Figure 1). On the other hand, when multiple cloud types are present in a grid (e.g., Grids 7 and 8 in Figure 1), they should share the same environment. This argument on spatial averaging over a region is also applicable to temporal averaging for a given grid because a cloud type may be present in 1 day but not in other days. That is, the time‐averaged, for example, the monthly mean, clear‐sky flux may be slightly different from that averaged only over the days when a cloud type is present. In another word, the regionally averaged CRE of a given cloud type should be computed from in‐cloud radiative fluxes of the given type and the clear‐sky fluxes in the vicinity of that cloud type not clear‐sky fluxes from distant environments (i.e., Grids 1, 2, 6 and 9 in Figure 1) that could not produce that cloud type. These factors motivate us to derive a new expression for regionally averaged clear‐sky flux (see Section 2.2).

In this study, we will use two clear‐sky flux expressions to calculate regionally averaged CREs and CREs by cloud type, one considering the influence of immediate environment of clouds and the other without. The latter uses the regionally averaged clear‐sky fluxes that are identical for all cloud types. We choose five convectively active regions in the Tropics, with two over land (Africa and Amazon) and three over ocean (eastern and western Pacific and Atlantic), in addition to the entire tropical region. Following Takahashi et al. (2017), these five tropical regions are defined as: Africa (0–35°E, 12°S–24°N), Amazon (280–325°E, 15°S–10°N), tropical western Pacific (TWP) (90–170°E, 15°S–15°N), eastern Pacific intertropical convergence zone (ITCZ) (180–280°E, 0–12°N), and Atlantic ITCZ (310–345°E, 0–12°N). Note that both longitudinal (35°–100°) and latitudinal (12°–35°) ranges of these regions vary greatly. The 25°S–25°N latitudinal belt is chosen to represent the entire tropical region. In addition to comparison between the results obtained from two clear‐sky flux expressions for calculating the CREs by cloud type, we will also examine the similarity and differences in CREs and in‐cloud and clear‐sky radiative fluxes for the five chosen regions particularly the differences between land and oceanic regions.

The rest of the paper is organized as follows. Section 2 describes the FBCT data set and the methodologies for calculating the CREs by cloud type. Results are presented in Section 3 including the tropical mean CREs by cloud type and the CRE and flux differences by cloud type for the five chosen regions from those of the tropical mean. Summary and discussions are given in Section 4.

## 2 Data Set and Methodology

### 2.1 FluxByCldTyp (FBCT) Data Set

In this study, we use the daily 1° × 1° daytime CERES Aqua FBCT data between July 2002 and June 2021. The data volume is ∼6 GB per year. The CERES FBCT data set provides either combined or separated Terra and Aqua daily (or monthly) daytime‐averaged cloud properties and radiative fluxes over 1° × 1° grid stratified by effective cloud (“radiating” top) pressure ($p_{c}$) and cloud optical depth (τ) bins (Eitzen et al., 2017; Sun et al., 2022). A cloud type is described by a $p_{c\;}$‐ τ pair using the Moderate Resolution Imaging Spectrometer (MODIS) pixel (2 × 2 km^2^) data. In practice, only 42 discrete cloud types are chosen using seven $p_{c}$ bins and six τ bins, plus a cloud‐free bin, following the ISCCP definition of cloud types (Rossow & Schiffer, 1999). Cloud‐free areal fraction is computed from 1 minus the sum of cloud fractions over 42 cloud types. Microphysical and macrophysical properties of these cloud types are derived directly from the CERES cloud retrieval with the MODIS pixel data (Minnis et al., 2011, 2021). The algorithm for obtaining cloud type‐sorted radiative fluxes and clear‐sky fluxes utilizes empirically derived narrowband to broadband coefficients to obtain broadband radiances, conversion of the mean broadband radiances to the subfootprint fluxes using the CERES angular distribution models (ADMs) and verification against the “observed” footprint flux (Loeb et al., 2018, 2020). Note that the average CERES footprint size at nadir on Aqua is approximately 20 km in diameter. Fluxes of each of the single‐scene footprints and subfootprint cloud layers are assigned to $p_{c\;}$– τ cloud types and the areal extents of single‐scene footprints and subfootprint cloud layers are used to obtain cloud‐type averaged fluxes for 1° × 1° grid, where single‐scene footprints refer to overcast footprints with a single cloud layer while subfootprints refer to partially cloudy parts of footprints with one or two cloud layers (see Figure 15 of Minnis et al., 2011 and Figure 2 of Sun et al., 2022). Instantaneous shortwave fluxes including clear‐sky fluxes are converted to equivalent daily mean fluxes by the method explained in Sun et al. (2022).

### 2.2 Clear‐Sky Flux Expressions for Calculating Cloud‐Type Mean Cloud Radiative Effects

In this section, we derive a CRE expression that considers the influence of immediate environment of clouds. Traditionally, CRE over an arbitrary area or a model grid box is defined as the difference in radiative flux, defined as positive for upward flux, between the clear and all skies, that is,

(1) $$\text{CRE}=F_{\text{clr}}-F_{\text{all}}$$

where subscripts clr and all denote clear skies and all skies, respectively (Ramanathan et al., 1989). If both in‐cloud and clear‐sky fluxes are horizontally uniform in their respective areas, CRE is equal to the difference between the two fluxes multiplied by cloud fraction, that is,

(2) $$\text{CRE}=F_{\text{clr}}-\left[{\mathrm{aF}}_{\text{cld}}+\left(1-a\right)\;F_{\text{clr}}\right]=a\left(F_{\text{clr}}-F_{\text{cld}}\right)$$

where a is cloud fraction and subscript cld denotes cloudy skies or “in‐cloud.” The cloud‐type mean CRE, that is, ${\text{CRE}}_{\text{cld}}$, can be similarly defined as in Equation 1 except for replacing all sky flux ($F_{\text{all}}$) by averaged in‐cloud flux, $F_{\text{cld}}$, that is,

(3) $${\text{CRE}}_{\text{cld},j}=F_{\text{clr}}-F_{\text{cld},j}$$

where subscript *j* denotes a cloud type. Note that Equation 3 is identical to Equation 2 if cloud fraction, a *,* is 1.

For computing cloud‐type mean CREs over a large region (composed of many FBCT grids) such as the tropical western Pacific (TWP) in a given day, the simple difference between the averaged $F_{\text{clr}}$ and $F_{\text{cld}}$ over all 1° × 1° grids within a region using Equation 3 yields CRE values with wrong signs, that is, positive SW CRE and negative LW CRE for a few infrequently occurring cloud types, that is, clouds reflect less solar radiation and emit more infrared radiation relative to the regionally averaged clear skies. This result suggests that the regionally averaged clear‐sky flux does not represent that of the adjacent clear skies near clouds of a particular type. A similar argument can be made for the temporal averaging. To overcome this problem, the cloud‐type mean CRE Equation 3 multiplied by cloud fraction, $a_{j}{\times \text{CRE}}_{\text{cld},j}$, is computed. This means that clear‐sky fluxes are used in the CRE calculation only when a particular cloud type exists in a 1° × 1° grid. That is, $a_{j}=0$ ensures that the clear‐sky flux over a grid, where cloud type *j* is not present, does not contribute to the regionally averaged CRE by cloud type. Figure 1 presents a schematic for illustrating how the proposed expression calculates the regional mean clear‐sky radiative fluxes compared to the expression using the regionally averaged clear‐sky fluxes. Grids 1, 2, 6, and 9 in Figure 1 do not contribute to the regional mean clear‐sky radiative fluxes with the new expression.

Regionally averaged CRE by cloud type, ${\bar{\text{CRE}}}_{\text{cld},j}$, is the sum of this cloud fraction‐weighted CRE over all 1° × 1° grids within a region divided by cloud fraction of cloud type *j* over this region. Mathematically, in a discrete form,

(4) $${\bar{\text{CRE}}}_{\text{cld},j}=\frac{1}{N}\sum_{i=1}^{N}\left[({F_{\text{clr},i}-F}_{\text{cld},i,j}\right.)a_{i,j}]\;/\;{\bar{a}}_{j}$$

where *N* is the total number of 1° × 1° grids over the region, $a_{i,j}$ is cloud fraction of a cloud type over *i*th grid and ${\bar{a}}_{j}=\frac{1}{N}\sum_{i=1}^{N}a_{i,j}$ regionally averaged cloud fraction. Note that a second index, *i*, is added on the right‐hand side of Equation 4 to represent *i*th grid. The right‐hand side of Equation 4 can be split into two parts. The first part is the averaged in‐cloud radiative flux over all grids:

(5) $${\bar{F}}_{\text{cld},j}=\frac{1}{N}\sum_{i=1}^{N}\left[F_{\text{cld},i,j}a_{i,j}\right]\;/\;{\bar{a}}_{j}$$

The second part is the cloud fraction‐weighted clear‐sky flux, that is,

(6) $${\bar{F}}_{\text{clr},j}=\frac{1}{N}\sum_{i=1}^{N}\left[F_{\text{clr},i}\;a_{i,j}\right]\;/\;{\bar{a}}_{j}$$

In Equation 6, the clear‐sky flux on the *i*th grid does not contribute to the regionally averaged clear‐sky flux of cloud type *j* if cloud fraction of cloud type *j* on this grid is zero (e.g., Grids 1, 2, 6, and 9 in Figure 1). On the other hand, without considering the influence of immediate environment of clouds, the clear‐sky fluxes of individual cloud types are independent upon cloud type, that is,

(7) $${\bar{F}}_{\text{clr}}=\sum_{i=1}^{N}\left(1-\sum_{j=1}^{42}a_{i,j}\right)F_{\text{clr},i}\;/\sum_{i=1}^{N}\left(1-\sum_{j=1}^{42}a_{i,j}\right)$$

This formula is identical to Equation 6 if clear‐sky flux is horizontally uniform. As will be shown in Section 3, the difference between Equations 6 and 7 can significantly impact the regionally averaged CREs and CREs by cloud type. In this study, Expression 1 uses Equation 7 to calculate regionally averaged clear‐sky flux, whereas Expression 2 uses Equation 6 to do so. Expression 1 has been used in several studies but with the monthly FBCT data (Raghuraman et al., 2023, 2024; Scotts et al., 2020).

Once the averaged CRE by cloud type over a region is known, regionally averaged CRE can be expressed as follows. That is, CREs of all 42 cloud types weighted by their respective cloud fractions are summed up:

(8) $$\text{CRE}=\sum_{j=1}^{42}{\bar{a}}_{j}{\bar{\text{CRE}}}_{\text{cld},j}=\frac{1}{N}\sum_{j=1}^{42}\sum_{i=1}^{N}\left[\left({F_{\text{clr},i}-F}_{\text{cld},i,j}\right)a_{i,j}\right]$$

The horizontal nonuniformity in both cloudy and clear‐sky fluxes over a large region are expressed in this formula. This means that nonlinearity through the multiplication of cloud fraction with radiative fluxes is retained in the CRE calculation using high‐resolution cloud and radiative flux data from the CERES FBCT product.

Lastly, before the computation outlined above is performed, missing clear‐sky fluxes (or with default values) over 1° × 1° grids within a region are filled with valid values from nearest grids, which are justified because clear‐sky fluxes are much more spatially uniform than their cloudy sky counterparts. This is a necessary step in the CRE computation because clear‐sky fluxes for overcast conditions are not available in the FBCT data product. This procedure does not impact the conclusions obtained from this study because the percentage of such grids is very small.

## 3 Results

### 3.1 Tropical Mean Cloud Radiative Effects by Cloud Type

**Figure 2:**
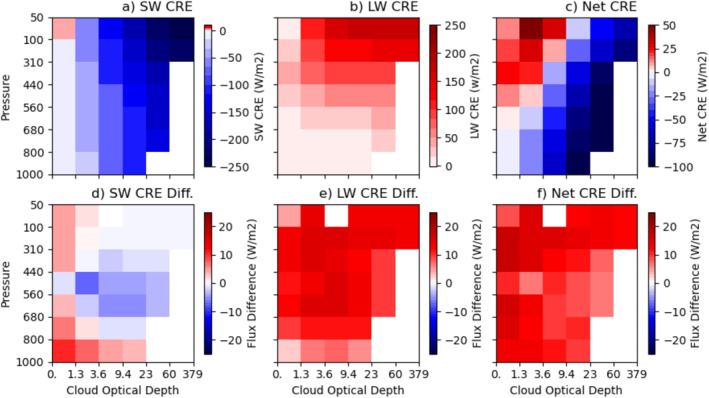
The cloud‐type ($p_{c}$‐ τ) mean shortwave cloud radiative effect, SW CRE (a), longwave cloud radiative effect, LW CRE (b), and net cloud radiative effect, Net CRE (c) for the entire tropical region averaged over 19 years. The cloud‐type mean difference (Expression 1–Expression 2) in SW CRE (d), LW CRE (e), and Net CRE (f) for the entire tropical region is calculated from clear‐sky fluxes with (Expression 2) from that without (Expression 1) considering the influence of immediate environment of clouds. The cloud‐type mean CRE difference is due to different definitions of regionally averaged clear‐sky fluxes. The entire tropical region is defined as the latitudinal belts between 25°S and 25°N.

Figures 2a–2c show the tropical‐mean shortwave (SW), longwave (LW), and net cloud radiative effects (CREs) by cloud type, respectively. These are computed with Expression 2 proposed in Section 2.2 that considers the influence of immediate environment of clouds. That is, ${\bar{\text{CRE}}}_{\text{cld},j}={\bar{F}}_{\text{clr},j}-{\bar{F}}_{\text{cld},j}$. The results are averaged over 19‐year data record, which means that the results include influences of immediate environment of clouds from both spatial and temporal averaging. The tropical‐mean CREs by cloud type provide a reference for comparing differences among the five chosen regions to be discussed later. In all figures presented in this study, cloud types with less than 0.05% area fractions are drawn with the white color to avoid weak statistics. Also, any CRE/flux difference with values between −1 and +1 W m^−2^ (two lightest colors in plots) is within retrieval uncertainties and should be ignored.

The tropical‐mean SW CRE increase monotonically with the increase of τ and the decrease of $p_{c}$, ranging from slightly positive (1.5 W m^−2^) for a thin cirrus cloud type to −247.5 W m^−2^ for a deep convective cloud type (Figure 2a). The tropical‐mean LW CRE has similar dependencies except for weaker variations with τ but stronger dependencies on $p_{c}$ with a range of 2.5–175.5 W m^−2^ (Figure 2b). Due to the opposite signs between SW and LW CREs, the tropical‐mean net CREs are small residues for most of the cloud types except for optically thick (τ > 9.4) low‐level cloud types (right and lower part of Figure 2c), which have relatively strong net cooling effects. On the other hand, the optically thin (τ ≤ 9.4), middle‐ and upper‐level clouds (left upper corner of Figure 2c) have net warming effects. The net CREs by cloud type vary from −120.8 to 50.9 W m^−2^. The number of cloud types with net cooling effects is twice of that with net warming effects (Figure 2c). It is not surprising that the tropical‐mean net CRE, which is the sum of cloud‐type mean net CREs, as shown in Figure 2c, weighted by cloud fractions (not shown), over all 42 cloud types, is negative over the Tropics. That is, clouds radiatively cool the planet over the Tropics, but the magnitude of cooling depends upon on the areal fractions of cloud types. For example, if thin anvil cloud types are a dominant contributor, cooling may be very small.

**Figure 3:**
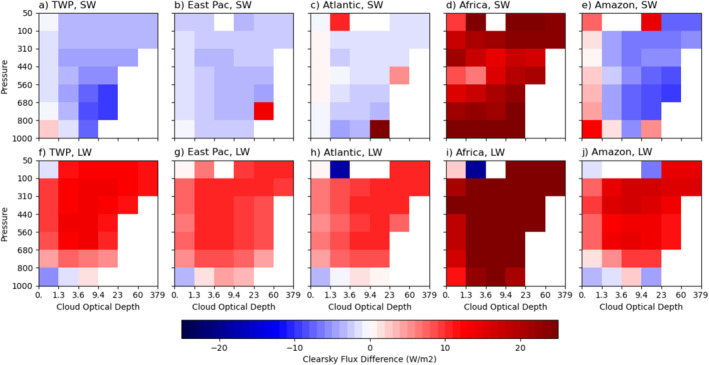
Panels (a–e) and (f–j) are respectively identical to Figures 2d and 2e except for Tropical Western Pacific (a, f), eastern Pacific (b, g) and Atlantic (c, h) intertropical convergence zones (ITCZs), Africa (d, i) and Amazonia (e, j) regions.

Figures 2d–2f show the SW, LW, and net CRE differences by cloud type between using clear‐sky flux Expression 1 (${\bar{F}}_{\text{clr}}$) and Expression 2 (${\bar{F}}_{\text{clr},j}$). These differences represent the amounts of influence of immediate environment of clouds on the CRE calculation, that is, the differences in clear‐sky fluxes, ${\bar{F}}_{\text{clr}}-{\bar{F}}_{\text{clr},j}$, between the two expressions because averaged in‐cloud radiative fluxes (${\bar{F}}_{\text{cld},j}$; Equation 5) used in both CREs are identical. Figures 2d–2f show that these differences are not trivial for some of the cloud types comparing to the magnitudes of CREs. For SW CRE differences, the signs can be either positive or negative (Figure 2d) ranging from −7.8 to 10.7 W m^−2^. Environments of optically thin (with τ≤ 1.3) cloud types and low‐level ($p_{c}\leq$ 800 hPa) cloud types reflect less solar radiation than the regionally averaged clear skies (${\bar{F}}_{\text{clr}}$). This is also seen over both Africa and Amazon (Figures 3d and 3e). However, the differences have opposite signs between the two regions for the rest of cloud types. These positive differences mean that the immediate environments of clouds reflect much less solar radiation for all cloud types of Africa (Figure 3d) than the regionally averaged clear skies, typically 20–30 Wm^−2^. Large contrasts in surface albedo are a plausible explanation. On the other hand, environments of middle‐level cloud types (440 < $p_{c}\leq$ 680 hPa and τ > 1.3) for the entire tropical region reflect more solar radiation (Figure 2d). This is mainly contributed from oceanic and Amazonia regions, typically 2 to 6 Wm^−2^ (Figures 3a–3c and 3e), where congestus clouds, whose environments are moister, are more prevalent.

For LW and net CRE differences of the entire tropical region, however, the sign is positive for all cloud types ranging from 2.9 to 15.8 W m^−2^ for LW CRE and 6.1–17.9 W m^−2^ for net CRE, respectively (Figures 2e and 2f). One of the reasons is the daytime temperature difference between the regionally averaged clear skies and the immediate environment of clouds over land. The daytime only measurements of the FBCT data (Sun et al., 2022) contribute to these large clear‐sky flux differences over Africa, typically 20–30 Wm^−2^, up to 40–50 Wm^−2^, compared to data with complete diurnal cycles. The oceanic and Amazonia regions also show positive differences, typically 7–10 Wm^−2^ (Figures 3f–3h and 3j). This suggests that the regionally averaged clear skies are drier and warmer than the near‐cloud environments.

### 3.2 Regionally Averaged Cloud Radiative Effects by Cloud Type

**Figure 4:**
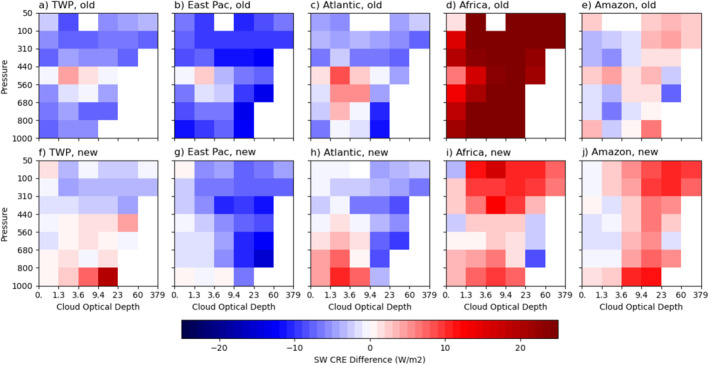
The cloud‐type ($p_{c}$‐ τ) mean difference in shortwave cloud radiative effect (SW CRE) from the entire tropical region calculated without (top row; “old” expression of clear‐sky flux or Expression 1) and with (bottom row; “new” expression of clear‐sky flux or Expression 2) considering the influence of immediate environment of clouds for Tropical Western Pacific (a, f), eastern Pacific (b, g) and Atlantic (c, h) intertropical convergence zones (ITCZs), equatorial Africa (d, i) and Amazonia (e, j) regions. See texts for the latitudinal and longitudinal bounds of these regions.

**Figure 5:**
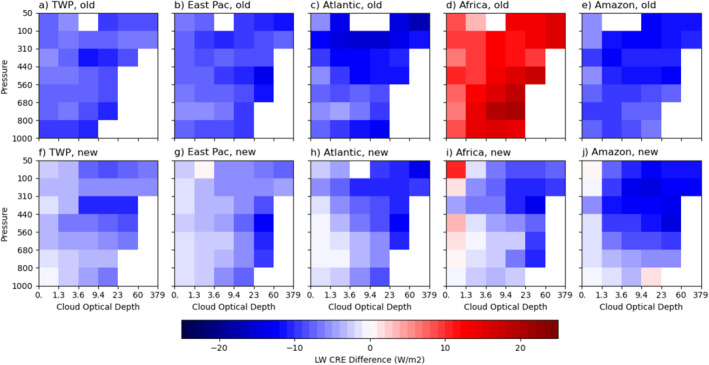
As in Figure 4, except for longwave cloud radiative effect difference from the entire tropical region.

**Figure 6:**
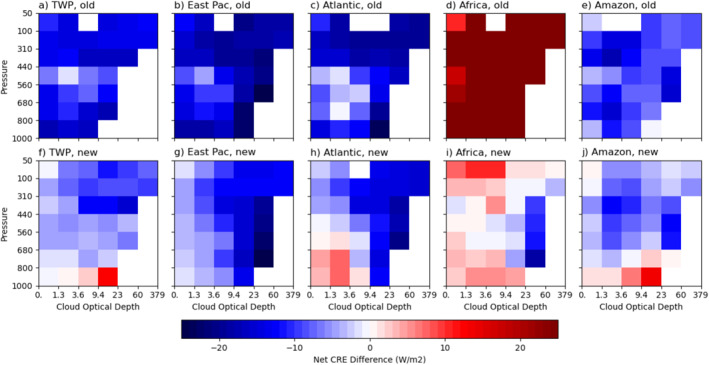
As in Figure 4, except for net cloud radiative effect difference from the entire tropical region.

To see the differences among the five chosen regions more clearly, we subtract the cloud‐type mean values of CREs by those of the entire tropical region (including all lands and ocean between 25°S and 25°N) as presented in Figure 2. Figures 4, 5, 6 show the SW, LW, and net CRE differences for five regions from the tropical means, respectively. The top rows of these figures show results obtained with Expression 1 (with regionally averaged clear‐sky fluxes) and the bottom rows with Expression 2 (with considering the influence of immediate environment of clouds). Note that the tropical means used in the top rows are the sum of the top and bottom rows of Figure 2. As discussed below, there are stronger similarities in CREs by cloud type among the three oceanic regions compared to the two land regions and the differences in CREs among the regions are much larger with Expression 1 than with Expression 2, as detailed below.

With Expression 1, SW cloud cooling over Africa is much smaller than the tropical mean by up to 36.6 W m^−2^ (Figure 4d). The three oceanic regions have stronger SW cloud cooling than the tropical mean for most cloud types except for a few middle‐level cloud types with moderate τ (Figures 4a–4c), whereas Amazon is the closest to the tropical mean for nearly all cloud types (Figure 4e). With Expression 2, SW cloud cooling over Africa increase by about 20 W m^−2^ for most cloud types (Figure 4i), but its SW cloud cooling is still weaker than the tropical mean for upper‐level cloud types by up to 16.5 W m^−2^ and the lowest‐level cloud types by less than 10 W m^−2^. Over Amazon, SW cloud cooling is also weaker than the tropical mean by less than 10 W m^−2^ for all optically moderate/thick (τ > 3.6) cloud types but close to the tropical mean for optically thin cloud types (Figure 4j). In other words, SW cloud cooling over the two land regions resembles each other more (Figures 4i and 4j) compared to that calculated with Expression 1 (Figures 4d and 4e). This result suggests that the influences of immediate environment for most cloud types are opposite between the two regions, owing to the different amounts of contrasts in moisture and possibly aerosols between clear areas without nearby clouds and the near‐cloud environments.

Comparing that with Expression 1 (Figures 4a–4c), SW cloud cooling from Expression 2 for the three oceanic regions (Figures 4f–4h) becomes closer to the tropical mean for optically thin and low/middle‐level cloud types but has more contrasts for some optically thick cloud types among the three regions, that is, eastern Pacific has the strongest cooling (Figure 4g), TWP is very close to the tropical mean for most cloud types (Figure 4e), whereas Atlantic is in between the other two regions except for more optically thin low‐level cloud types with less cooling (Figure 4h) than the tropical mean compared to TWP. It is interesting to point out that the SW warming relative to the tropical mean for some of the congestus cloud types with Expression 1 (Figures 4a–4c) is reduced with Expression 2 (Figures 4f–4h).

LW cloud warming effects calculated with Expression 2 (Figure 2b) are smaller than those with Expression 1 for the tropical mean attributed partly to changes in the LW CRE over land regions such as Africa (Figure 5d). The LW CRE differences become very small among the five chosen regions (Figure 5) despite much greater differences in both clear and cloudy sky fluxes among the regions, as shown later. Another noticeable result is that LW CRE over Africa is reduced by 20–25 W m^−2^ for some cloud types (Figures 5d and 5i). This is related to that the immediate environments of clouds are more humid and probably cooler. Both factors can reduce clear sky outgoing infrared emission, especially for all cloud types over Africa and optically thin cloud types and low‐level cloud types over the oceanic regions (5f–j).

Compared to SW CRE (Figure 4), net CRE differences from the tropical mean are more negative (Figure 6) because LW CRE differences are negative for all regions (Figure 5) except for Africa using Expression 1 (Figure 6d). In this case, the net CRE difference (Figure 6d) becomes more positive than those of SW CRE differences (Figure 4d) ranging from 10.2 to 52.3 W m^−2^. With Expression 2, the magnitudes of net cloud cooling effects are reduced for most cloud types of the three oceanic and Amazon regions. The reason for this result is the same as that of LW CRE differences discussed earlier. Over Africa, the net cloud cooling is increased but still less than the tropical mean for most of the cloud types ranging from −18.9 to 10.4 W m^−2^ for the net CRE differences from the tropical mean. However, the regionally averaged net cloud cooling (Equation 8), as discussed in Section 3.4, indicates that clouds also have net cooling effects as in other four regions instead of net warming effects using Expression 1.

### 3.3 Regionally Averaged In‐Cloud and Clear‐Sky Radiative Fluxes by Cloud Type

**Figure 7:**
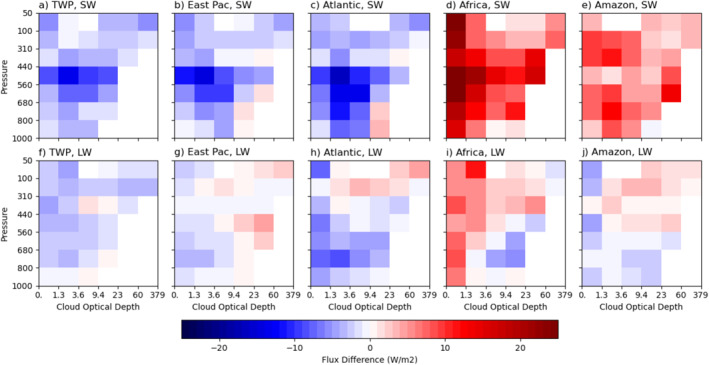
The cloud‐type ($p_{c}$‐ τ) mean difference in in‐cloud shortwave radiative flux (top row) and longwave radiative flux (bottom row) from the entire tropical region for Tropical Western Pacific (a, f), eastern Pacific (b, g) and Atlantic (c, h) intertropical convergence zones (ITCZs), equatorial Africa (d, i) and Amazonia (e, j) regions. See texts for the latitudinal and longitudinal bounds of these regions.

In this section, we focus on in‐cloud and cloud fraction‐weighted clear‐sky radiative fluxes, which are used to calculate the CREs (see Equations (4), (5), (6)) using Expression 2, because both fluxes can contribute to the CRE differences among the regions. We first examine the averaged in‐cloud SW and LW radiative flux differences by cloud type from the tropical mean (Figure 7). All cloud types over the two land regions reflect more solar radiation than the tropical mean by up to 13.9 W m^−2^ over Amazon and 36.2 W m^−2^ over Africa, respectively (Figures 7d and 7e). The optically thin cloud types over Africa (τ≤ 3.6) stand out as the brightest relative to the tropical mean (Figure 7d), which could be due to the reflection from the surface owing to its high surface albedo. These clouds may be optically thin enough to let most of the surface reflection to pass through. The optically thicker clouds are, however, slightly brighter than the tropical mean over these land regions owing to the decreased influence of surface reflection as τ increases.

The three oceanic regions have similar averaged in‐cloud SW radiative flux differences by cloud type from the tropical mean. Most of the cloud types reflect less solar radiation than the tropical mean up to −13.5, −14.7, and −16.6 W m^−2^ for TWP, eastern Pacific, and Atlantic regions, respectively (Figures 7a–7c), which means that cloud cooling effects are stronger than the tropical mean if the clear‐sky flux is kept the same as the tropical mean. Middle‐level (congestus) cloud types (440 < $p_{c}\leq$ 680 hPa) stand out as the dimmest, which may also be attributed to the high moisture above the tops of congestus clouds. The rest of cloud types are only slightly dimmer than the tropical mean. The ranges of variations in SW flux differences among all cloud types are 13.1, 16.4, 20.2, 35.2, and 14.1 W m^−2^ for TWP, eastern Pacific, Atlantic, Africa, and Amazon, respectively.

The averaged in‐cloud LW radiative flux differences from the tropical mean have smaller magnitudes (Figures 7f–7j) and more similar among the five regions compared to those of in‐cloud SW radiative flux differences (Figures 7a–7e). Most of the cloud types are very close to the tropical means particularly over TWP, eastern Pacific, and Amazon regions. A few optically thin (τ≤ 3.6) and low‐level cloud types emit less infrared radiation for Atlantic (up to −8.2 W m^−2^) but more infrared radiation for Africa (up to 11.5 W m^−2^) than the tropical mean. The latter is due to the high surface infrared emission owing to high daytime temperature, which can contribute to the emission of optically thin cloud types (τ≤ 3.6). The ranges of variations in LW flux differences among all cloud types are 5.6, 7.4, 12.8, 17.1, and 7.7 W m^−2^ for TWP, eastern Pacific, Atlantic, Africa, and Amazon, respectively.

**Figure 8:**
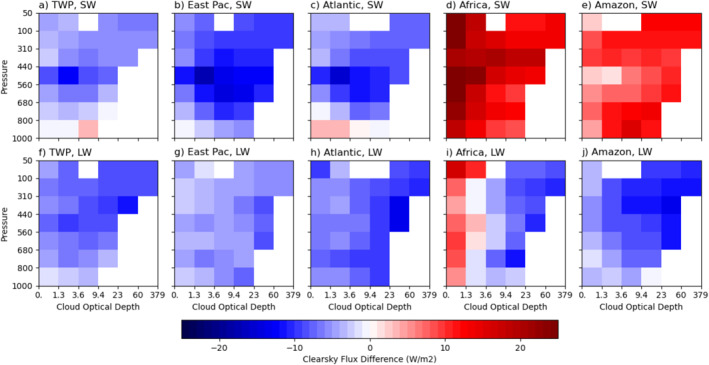
As in Figure 7, except for clear‐sky shortwave and longwave radiative flux differences from the entire tropical region.

The averaged differences of clear‐sky SW and LW radiative flux by cloud type (see Equation 6) from the tropical mean are shown in Figure 8. Note that subtraction of these fluxes by the averaged in‐cloud fluxes (Figure 7) yields the SW CRE (Figures 4f–4j) and LW CRE (Figures 5f–5j). The clear‐sky SW flux is more similar between the three oceanic regions than the two land regions (Figures 8a–8e) as in the SW CRE differences (Figures 4f–4j) and in‐cloud SW radiative flux differences (Figures 7a–7e), whereas the clear‐sky LW flux differences (Figures 8f–8j) are rather similar among the five regions as in the LW CRE differences (Figures 5f–5j) except for optically thin cloud types over Africa. In general, the magnitudes of clear‐sky SW and LW flux variations from one cloud type to another are not small. The ranges of variations in clear‐sky SW flux are compared to those of in‐cloud SW flux, that is, 16.8, 16.7, 18.9, 24.5, and 14.8 W m^−2^ for TWP, eastern Pacific, Atlantic, Africa, and Amazon, respectively, but those for clear‐sky LW flux are much larger than those of in‐cloud LW flux (10.6, 6.6, 9.6, 28.2, and 12.4 W m^−2^ for the same five regions). This result suggests that use of cloud‐type independent regionally averaged clear‐sky flux (see Equation 7) can result in large errors in computing the CREs by cloud type and the regionally averaged CREs. The latter will be discussed in Section 3.4.

The greater differences among the cloud types in both clear‐sky SW and LW fluxes over land are related to the greater impact of land surface on optically thin cloud types compared to optically thick cloud types. For example, large surface albedo over land can impact the clear‐sky SW fluxes, whereas higher surface temperature can impact the clear‐sky LW fluxes if clouds are optically thin enough. These influences from land surface are diminished as cloud optical thickness increases as more humid environments favor deeper convection and optically thicker cloud types. As surface evaporation is reduced with increasing moisture, sensible heat flux is also reduced, which lowers the environmental temperature. More moisture also reduces clear sky infrared emission for optically thick cloud types over ocean. The moisture contrast can also be used to explain the contrast between the two land regions owing to much moister environment over Amazon than over Africa.

### 3.4 Regionally Averaged Cloud Radiative Effects

Regionally averaged CREs depend upon the cloud fraction of cloud types in a region and their relative dominance (Table 1). The regionally averaged SW CRE for the entire tropical region is −39.6 W m^−2^ from both clear‐sky flux expressions. This means that there must be compensations occurring elsewhere outside of the five chosen regions particularly over the land regions such as Africa. Less LW cloud warming and stronger net cooling, by 5.1 W m^−2^ are obtained with Expression 2, resulting in a net cloud cooling of −16.3 W m^−2^ instead of −11.2 W m^−2^. This result is expected from LW and net CRE differences by cloud type shown in Figures 2e and 2f.

**Table 1:** Cloud Fraction (of All Cloud Types Combined) and Cloud Radiative Effects (CREs) Without (Expression 1) and With (Expression 2) the Influence of Immediate Environment of Clouds for the Entire Tropical Region and Five Convectively Active Regions (Tropical Western Pacific, TWP; Eastern Pacific and Atlantic Intertropical Convergence Zones, Africa and Amazon)

Region	Cloud fraction	CRE from Expression 1			CRE from Expression 2		
		SW	LW	Net	SW	LW	Net
Entire Tropics	0.596	−39.6	28.5	−11.1	−39.6	23.4	−16.3
TWP	0.730	−57.9	46.6	−11.3	−55.6	39.9	−15.7
E Pacific	0.688	−55.1	30.2	−24.9	−52.5	27.1	−25.4
Atlantic	0.648	−47.3	26.7	−20.6	−44.4	25.7	−18.7
Africa	0.513	−25.0	34.4	+9.4	−34.7	21.8	−12.9
Amazon	0.693	−59.2	32.8	−26.6	−54.2	28.5	−25.7

*Note.* Shortwave (SW), Longwave (LW) and Net CREs are Compared. Unit is Wm^−2^.

The SW CREs for the three oceanic regions differ by ∼10 W m^−2^ with SW cloud cooling of −47.3 to −57.9 W m^−2^ and −44.4 to −55.6 W m^−2^ from the two clear‐sky flux expressions, respectively. The large magnitudes are associated with the TWP region where total cloud fraction is the highest (Table 1) and upper‐level anvils dominate (not shown). The differences between the two clear‐sky flux expressions are only 2.3, 2.6, and 2.9 W m^−2^ for these three regions. The LW CRE differences for the three regions differ by twice of those of SW CRE (26.7–46.6 for Expression 1 and 25.7 to 39.9 for Expression 2, respectively). Table 1 shows that the influence of the immediate environment of clouds is to reduce both the SW cloud cooling and LW cloud warming over the oceanic regions. It is noted that the LW CRE difference is the greatest over TWP (6.7 W m^−2^) compared to 1.0 W m^−2^ for Atlantic and 3.1 W m^−2^ for eastern Pacific, respectively. This is probably related to the influence of nearby landmasses and warm SST areas associated with large‐scale divergence, that is, the areas without convection. The net cloud cooling is, however, the smallest over TWP (−11.3 or −15.7 W m^−2^) but the largest over eastern Pacific (∼−25 W m^−2^ from either expression), which is attributed to the larger cancellation of warming of thin anvils with cooling of thick anvils over TWP compared to over eastern Pacific where fractions of anvil cloud types are approximately half of those over TWP. The net cloud cooling increases by 4.4 W m^−2^ for TWP, 0.5 W m^−2^ for eastern Pacific but decreases by 1.9 W m^−2^ for Atlantic using Expression 2, relative to Expression 1.

For the two land regions, considering the influence of immediate environment of clouds reduces the LW cloud warming by 12.6 W m^−2^ for Africa compared to 4.3 W m^−2^ for Amazon. It also increases the SW cloud cooling by 9.7 W m^−2^ for Africa compared to 5.0 W m^−2^ for Amazon. The net cloud effects change from a net warming of 9.4 W m^−2^ to a net cooling of −12.9 W m^−2^ for Africa. The net cloud cooling agrees with the other four regions. However, the net cloud cooling is only slightly changed from −26.6 to −25.7 W m^−2^ for Amazon. These results are consistent with those of CREs by cloud type presented in Section 3.2 and point to the greatest influence of immediate environment of clouds over Africa among the five regions.

## 4 Summary and Discussions

This study has examined how much choice of clear‐sky fluxes impacts regionally averaged cloud radiative effects (CREs) and CREs by cloud type. The region is defined as a domain that is much larger than the grid size of the FBCT data (1° × 1°) used in this study. CREs by cloud type for five tropical convectively active regions and the entire tropical region are calculated from the differences between the regionally averaged clear‐sky fluxes (same for all cloud types; Expression 1) and in‐cloud radiative fluxes and those between the cloud fraction‐weighted clear‐sky fluxes (Expression 2), which is cloud‐type dependent and in‐cloud radiative fluxes. These two expressions produce identical CREs by cloud type if clear‐sky fluxes over the entire region are horizontally uniform. The second expression does not allow any contribution of clear‐sky flux in a grid (1° × 1°) to regionally averaged CREs when a cloud type is absent in this grid. In another word, when a grid is 100% cloud free, its radiative fluxes are used in regionally averaged CRE calculation with the first expression but not the second expression. The five chosen regions are Africa, Amazon, eastern and western Pacific, and Atlantic ITCZ. Nineteen years of high‐resolution CERES satellite data‐derived fluxes by cloud type (FBCT) product (Sun et al., 2022) are utilized. Forty‐two cloud types are classified using the joint cloud‐top pressure ($p_{c\;}$) and cloud optical depth (τ) distribution (Rossow & Schiffer, 1999) plus cloud free bin. Missing clear‐sky fluxes over 1° × 1° grids within a region are filled with valid values from nearest grids before CREs are computed.

For the entire tropical region, both SW (ranging from 1.5 to −247.5 Wm^−2^) and LW (2.5–175.5 Wm^−2^) CREs by cloud type vary with both $p_{c\;}$ and τ with net CREs varying from −120.8 to 50.9 Wm^−2^. The number of cloud types with net cooling effects is twice of that with net warming effects. The differences in cloud‐type mean CRE between Expression 2 and Expression 1 range from −7.8 to 10.7 Wm^−2^ for shortwave (SW), 2.9 to 15.8 Wm^−2^ for longwave (LW), and 6.1 to 17.9 Wm^−2^ for net, respectively. The oceanic and Amazonia regions have negative (positive) values in SW (LW) CRE differences typically 2–6 Wm^−2^ in SW but 7–10 Wm^−2^ in LW, whereas Africa has positive values in both SW and LW CREs (typically 20–30 Wm^−2^, up to 40–50 Wm^−2^). Large contrasts in surface albedo and daytime temperature between clear skies away from clouds and near clouds over Africa are the plausible causes.

For the regionally averaged CREs, that is, sum of cloud‐type mean CREs weighted by cloud fractions over 42 cloud types, the influence of immediate environment of clouds reduces the LW cloud warming (from 1.0 Wm^−2^ for Atlantic to 4.3 Wm^−2^ for Amazon) and the SW cloud cooling (from −2.3 Wm^−2^ for TWP to −5.0 Wm^−2^ for Amazon) in three oceanic and Amazonia regions. But, it increases the SW cloud cooling for Africa by −9.7 Wm^−2^ and reduces the LW cloud warming by 12.6 Wm^−2^ there. This results in a change of net cloud effects from warming (9.4 Wm^−2^) to cooling (−12.9 Wm^−2^) for Africa, which is more physically reasonable and in agreement with the other four regions and the entire tropical region. The Amazonia region resembles the three oceanic regions more than its fellow land region except with slightly larger reductions in both SW (−5.0 Wm^−2^) and LW (4.3 Wm^−2^) CREs. This is not surprising due to the large contrasts in land temperature and moisture in the atmosphere between the two land regions. Compared to eastern Pacific (−0.5 Wm^−2^), Atlantic (+1.9 Wm^−2^), and Amazon (+0.9 Wm^−2^), TWP has a relatively large increase in net cloud cooling (from −11.3 to −15.7 Wm^−2^ or −4.4 Wm^−2^) due to a greater reduction in LW cloud warming (6.7 Wm^−2^) that is related to its influence of nearby landmasses and the high SSTs. For the entire tropical region, SW CRE is not impacted, but LW cloud warming is reduced by 5.1 Wm^−2^, resulting in an increase in equal amount of net cloud cooling. This is a significant amount of enhanced net cloud cooling effects when the influence of immediate environment of clouds is considered.

Because the five chosen regions are all convectively active, some similarities in SW, LW, and net CREs, and in‐cloud and clear‐sky SW and LW radiative FBCT among the regions are expected. To facilitate the comparisons, the differences in these quantities from their respective tropical means using the two clear‐sky flux expressions are examined. With the second expression, LW CREs are similar among the regions including Africa (except for a few optically thin cloud types) and the differences from the tropical means are very small for optically thin and low‐level cloud types. This is also the case for SW and net CREs of these cloud types plus stronger resemblance between the two land regions for other cloud types due to large reduction in LW cloud warming and increase of SW cloud cooling over Africa. For in‐cloud and clear‐sky SW and LW radiative fluxes, the three oceanic regions are more like each other than the two land regions are but both in‐cloud and clear‐sky LW radiative fluxes over Amazon resemble their oceanic counterparts.

What are the implications of these findings for observational and modeling studies?

1. Whether the fluxes of totally cloud‐free grids should be included or not in calculating the regional mean clear‐sky fluxes and thus CREs is an important issue. Obviously, such grids have zero CREs, but they can change the magnitudes of regionally averaged CREs by cloud type using the first expression. With the second expression, these grids are automatically excluded. Based upon the results presented in this study, the greatest differences in CREs resulting from these two clear‐sky flux expressions occur over the land regions, especially, over Africa. So, we propose to modify the first expression by calculating the regionally averaged clear‐sky fluxes by excluding the totally cloud‐free grids. It is expected that the differences in CREs between using the modified Expression 1 and Expression 2 may be greatly reduced for some of the cloud types, which can be tested from observational data.
2. If this modified Expression 1 discussed above reproduces reasonably accurate CREs compared to those from the second expression for most cloud types, it can be applicable to modeling studies with the cloud simulator approach (e.g., Eitzen et al., 2017). Eitzen et al. (2017) incorporated flux‐by‐cloud‐type simulators in climate models and compared the resulting cloud‐type fluxes with the prototype version of FBCT data presented by Sun et al. (2022). Errors in the fluxes can be separated from those in the frequency of occurrence of cloud types.
3. There are still gaps in CRE estimates from observations (Loeb et al., 2018, 2020; Ramanathan et al., 1989) and modeling studies due to slightly different definitions of CREs. Climate modelers use the differences between cloud‐free flux and all sky flux over grids with cloud fractions between 0 and 1. Observational studies are hampered by the accuracy of satellite cloud retrievals and the lack of truly clear pixels in overcast conditions over a large area. A radiative transfer model‐calculated clear‐sky flux with reanalysis data as input may provide a better representation of clear‐sky flux, which may be less reliance on satellite‐derived clear‐sky fluxes (Loeb et al., 2020).
4. Regional studies on cloud radiative impact to large‐scale circulations need to reexamined the impact using the second clear‐sky flux expression to provide a more accurate description of the differential radiative heating between clear and cloudy areas, for example, for understanding the mechanisms for convective self‐aggregation (Muller & Bony, 2015; Muller & Held, 2012; Pope et al., 2021; Xu et al., 2023).
5. Considering the recent analysis of cloud feedback, cloud masking, and cloud forcing using the monthly FBCT data (Raghuraman et al., 2023, 2024), it may be worth repeating the analyzes with CREs obtained from the second clear‐sky flux expression using the daily FBCT data instead of monthly FBCT data. This is because clear‐sky flux not in the vicinity of clouds may exert less influence on CREs and is likely associated with the effects of cloud masking and cloud forcing.

## Data Availability

The CERES FluxByCldTyp (FBCT) level‐3 data product is publicly available and can be accessed via CERES data portal (https://ceres.larc.nasa.gov/data/). The regionally averaged daily FBCT data are in the Zenodo database (Sun & Xu, 2024).

## References

[jgrd59913-bib-0001] Andersen, H. , Cermak, J. , Douglas, A. , Myers, T. A. , Nowack, P. , Stier, P. , et al. (2023). Sensitivities of cloud radiative effects to large‐scale meteorology and aerosols from global observations. Atmospheric Chemistry and Physics, 23(18), 10775–10794. 10.5194/acp-23-10775-2023

[jgrd59913-bib-0002] Boudala, F. S. , Milbrandt, J. A. , & Isaac, G. A. (2022). Evaluation of CanESM cloudiness, cloud type and cloud radiative forcing climatologies using the CALIPSO‐GOCCP and CERES datasets. Remote Sens. 2022, 14(15), 3668. 10.3390/rs14153668

[jgrd59913-bib-0003] Ceppi, P. , & Nowack, P. (2021). Observational evidence that cloud feedback amplifies global warming. Proceedings of the National Academy of Sciences of the United States of America, 118(30), e2026290118. 10.1073/pnas.2026290118 34282010 PMC8325336

[jgrd59913-bib-0004] Chen, T. , Rossow, W. B. , & Zhang, Y.‐C. (2000). Radiative effects of cloud‐type variations. Journal of Climate, 13(1), 264–286. 10.1175/1520-0442(2000)013<0264:reoctv>2.0.co;2

[jgrd59913-bib-0005] Cole, J. , Barker, H. W. , Loeb, N. G. , & von Salzen, K. (2011). Assessing simulated clouds and radiative fluxes using properties of clouds whose tops are exposed to space. Journal of Climate, 24(11), 2715–2727. 10.1175/2011JCLI3652.1

[jgrd59913-bib-0006] Eitzen, Z. A. , Su, W. , Xu, K.‐M. , Loeb, N. , Sun, M. , Doelling, D. R. , et al. (2017). Evaluation of a general circulation model by the CERES Flux‐by‐Cloud Type simulator. Journal of Geophysical Research: Atmospheres, 122(20), 10655–10668. 10.1002/2017JD027076 PMC8051178 33868884

[jgrd59913-bib-0007] Hartmann, D. L. , & Berry, S. E. (2017). The balanced radiative effect of tropical anvil clouds. Journal of Geophysical Research: Atmospheres, 122(9), 5003–5020. 10.1002/2017jd026460

[jgrd59913-bib-0008] Hartmann, D. L. , Okhert‐Bell, M. E. , & Michelson, M. L. (1992). The effect of cloud type on Earth's energy balance: Global analysis. Journal of Climate, 5(11), 1281–1304. 10.1175/1520-0442(1992)005<1281:teocto>2.0.co;2

[jgrd59913-bib-0009] Kiehl, J. T. (1994). On the observed near cancellation between longwave and shortwave cloud forcing in tropical regions. Journal of Climate, 7(4), 559–565. 10.1175/1520-0442(1994)007<0559:otoncb>2.0.co;2

[jgrd59913-bib-0010] Loeb, N. G. , Doelling, D. R. , Wang, H. , Su, W. , Nguyen, C. , Corbett, J. G. , et al. (2018). Clouds and the Earth’s Radiant Energy System (CERES) Energy Balanced and Filled (EBAF) Top‐Of‐Atmosphere (TOA) edition‐4.0 data product. Journal of Climate, 31(2), 895–918. 10.1175/jcli-d-17-0208.1

[jgrd59913-bib-0011] Loeb, N. G. , Rose, F. G. , Kato, S. , Rutan, D. A. , Su, W. , Wang, H. , et al. (2020). Toward a consistent definition between satellite and model clear‐sky radiative fluxes. Journal of Climate, 33(1), 61–75. 10.1175/jcli-d-19-0381.1

[jgrd59913-bib-0013] Minnis, P. , Sun‐Mack, S. , Young, D. F. , Heck, P. W. , Garber, D. P. , Chen, Y. , et al. (2011). CERES edition‐2 cloud property retrievals using TRMM VIRS and Terra and Aqua MODIS data—Part I: Algorithms. IEEE Transactions on Geoscience and Remote Sensing, 49(11), 4374–4400. 10.1109/TGRS.2011.2144601

[jgrd59913-bib-0014] Minnis, P. , Sun‐Mack, S. , Young, D. F. , Heck, P. W. , Garber, D. P. , Chen, Y. , et al. (2021). CERES MODIS cloud product retrievals for edition 4—Part I: Algorithm changes. IEEE Transactions on Geoscience and Remote Sensing, 59(4), 2744–2780. 10.1109/TGRS.2020.3008866

[jgrd59913-bib-0015] Muller, C. J. , & Bony, S. (2015). What favors convective aggregation and why? Geophysical Research Letters, 42(13), 5626–5634. 10.1002/2015GL064260

[jgrd59913-bib-0016] Muller, C. J. , & Held, I. M. (2012). Detailed investigation of the self‐aggregation of convection in cloud resolving simulations. Journal of the Atmospheric Sciences, 69(8), 2551–2565. 10.1175/JAS-D-11-0257.1

[jgrd59913-bib-0017] Najarian, H. , & Sakaeda, N. (2023). The influence of cloud types on cloud‐radiative forcing during DYNAMO/AMIE. Journal of Geophysical Research: Atmospheres, 128(8), e2022JD038006. 10.1029/2022JD038006

[jgrd59913-bib-0018] Pope, K. N. , Holloway, C. E. , Jones, T. R. , & Stein, T. H. M. (2021). Cloud‐radiation interactions and their contributions to convective self‐aggregation. Journal of Advances in Modeling Earth Systems, 13(9), e2021MS002535. 10.1029/2021MS002535

[jgrd59913-bib-0019] Raghuraman, S. P. , Medeiros, B. , & Gettelman, A. (2024). Observational quantification of tropical high cloud changes and feedbacks. Journal of Geophysical Research: Atmospheres, 129(7), e2023JD039364. 10.1029/2023JD039364

[jgrd59913-bib-0020] Raghuraman, S. P. , Paynter, D. , Menzel, R. , & Ramaswamy, V. (2023). Forcing, cloud feedbacks, cloud masking, and internal variability in the cloud radiative effect satellite record. Journal of Climate, 36(12), 4151–4167. 10.1175/jcli-d-22-0555.1

[jgrd59913-bib-0021] Ramanathan, V. , Cess, R. D. , Harrison, E. F. , Minnis, P. , Barkstrom, B. R. , Ahmad, E. , & Hartmann, D. (1989). Cloud‐radiative forcing and climate: Results from the Earth radiation budget experiment. Science, 243(4887), 57–63. 10.1126/science.243.4887.57 17780422

[jgrd59913-bib-0023] Randall, D. A. , Bony, S. , Colman, R. , Fichefet, T. , Fyfe, J. , Kattsove, V. , et al. (2007). Climate models and their evaluation. In Climate change 2007: The physical science basis. Contribution of working group I to the fourth assessment report of the intergovernmental panel on climate change (pp. 589–662). Cambridge University. Press.

[jgrd59913-bib-0022] Randall, D. A. , Coakley, J. A. Jr. , Fairall, C. W. , Kropfli, R. A. , & Lenschow, D. H. (1984). Outlook for research on subtropical marine stratiform clouds. Bulletin America Meteorology Social, 65(12), 1290–1301. 10.1175/1520-0477(1984)065<1290:ofrosm>2.0.co;2

[jgrd59913-bib-0024] Rossow, W. B. , & Schiffer, R. A. (1999). Advances in understanding clouds from ISCCP. Bulletin America Meteorology Social, 80(11), 2261–2287. 10.1175/1520-0477(1999)080<2261:aiucfi>2.0.co;2

[jgrd59913-bib-0025] Scott, R. C. , Myers, T. A. , Norris, J. R. , Zelinka, M. D. , Klein, S. A. , Sun, M. , & Doelling, D. R. (2020). Observed sensitivity of low‐cloud radiative effects to meteorological perturbations over the global oceans. Journal of Climate, 33(18), 7717–7734. 10.1175/JCLI-D-19-1028.1

[jgrd59913-bib-0026] Sohn, B. J. , Nakajima, T. , Satoh, M. , & Jang, H.‐S. (2010). Impact of different definitions of clear‐sky flux on the determination of longwave cloud radiative forcing: NICAM simulation results. Atmospheric Chemistry and Physics, 10(23), 11641–11646. 10.5194/acp-10-11641-2010

[jgrd59913-bib-0027] Stephens, G. L. (2005). Cloud feedbacks in the climate system: A critical review. Journal of Climate, 18(2), 237–273. 10.1175/JCLI-3243.1

[jgrd59913-bib-0028] Sun, M. , Doelling, D. R. , Loeb, N. G. , Scott, R. C. , Wilkins, J. , Nguyen, L. T. , & Mlynczak, P. (2022). Clouds and the Earth’s Radiant Energy System (CERES) FluxByCldTyp edition 4 data product. Journal of Atmospheric and Oceanic Technology, 39(3), 303–318. 10.1175/JTECH-D-21-0029.1

[jgrd59913-bib-0029] Sun, M. , & Xu, K.‐M. (2024). FluxByCldTyp Ed4 19 years of regional averaged data [Dataset]. Zenodo. 10.5281/zenodo.13527103

[jgrd59913-bib-0030] Takahashi, H. , Luo, Z. J. , & Stephens, G. L. (2017). Level of neutral buoyancy, deep convective outflow, and convective core: New perspectives based on 5 years of CloudSat data. Journal of Geophysical Research: Atmospheres, 122, 2958–2969. 10.1002/2016JD025969

[jgrd59913-bib-0031] Vial, J. , Dufresne, J.‐L. , & Bony, S. (2013). On the interpretation of inter‐model spread in CMIP5 climate sensitivity estimates. Climate Dynamics, 41(11–12), 3339–3362. 10.1007/s00382-013-1725-9

[jgrd59913-bib-0032] Wielicki, B. A. , Barkstrom, B. R. , Harrison, E. F. , Lee, R. B. , Smith, G. L. , & Cooper, J. E. (1996). Clouds and the Earth's Radiant Energy System (CERES): An Earth observing system experiment. Bulletin of the American Meteorological Society, 77(5), 853–868. 10.1175/1520-0477(1996)077<0853:catere>2.0.co;2

[jgrd59913-bib-0033] Xu, K.‐M. , Zhou, Y. , Sun, M. , Kato, S. , & Hu, Y. (2023). Observed cloud type‐sorted cloud property and radiative flux changes with the degree of convective aggregation from CERES data. Journal of Geophysical Research: Atmospheres, 128(19), e2023JD039152. 10.1029/2023JD039152

[jgrd59913-bib-0034] Yue, Q. , Kahn, B. H. , Fetzer, E. J. , Schreier, M. , Wong, S. , Chen, X. , & Huang, X. (2016). Observation‐based longwave cloud radiative kernels derived from the A‐Train. Journal of Climate, 29(6), 2023–2040. 10.1175/JCLI-D-15-0257.1

[jgrd59913-bib-0035] Zelinka, M. D. , Klein, S. A. , & Hartmann, D. L. (2012). Computing and partitioning cloud feedbacks using cloud property histograms. Part I: Cloud radiative kernels. Journal of Climate, 25(11), 3715–3735. 10.1175/jcli-d-11-00248.1

[jgrd59913-bib-0036] Zhou, C. , Zelinka, M. D. , Dessler, A. E. , & Yang, P. (2013). An analysis of the short‐term cloud feedback using Modis data. Journal of Climate, 26(13), 4803–4815. 10.1175/JCLI-D-12-00547.1

